# Topical administration of the secretome derived from human amniotic epithelial cells ameliorates psoriasis-like skin lesions in mice

**DOI:** 10.1186/s13287-022-03091-9

**Published:** 2022-08-03

**Authors:** Mengbo Yang, Lanqi Wang, Zhimin Chen, Weijie Hao, Qian You, Jianhua Lin, Jingzhi Tang, Xin Zhao, Wei-Qiang Gao, Huiming Xu

**Affiliations:** 1 State Key Laboratory of Oncogenes and Related Genes, Renji-MedX Clinical Stem Cell Research Center, Ren Ji Hospital, School of Medicine, Shanghai Jiao Tong University, Shanghai, 200127 China; 2 Department of Dermatology, Ren Ji Hospital, School of Medicine, Shanghai Jiao Tong University, Shanghai, 200127 China; 3 Department of Obstetrics and Gynecology, Ren Ji Hospital, School of Medicine, Shanghai Jiao Tong University, Shanghai, 200127 China; 4 Med-X Research Institute and School of Biological Medical Engineering, Shanghai Jiao Tong University, Shanghai, 200030 China

**Keywords:** Psoriasis, AEC-SC, Skin lesions, Skin inflammation, IL-1ra

## Abstract

**Background:**

Psoriasis is a chronic inflammatory skin disease. Tissue stem cells have exhibited a therapeutic effect on psoriatic mice. However, the therapeutic effect of topical administration of the secretome derived from tissue stem cells on psoriasis has not been reported.

**Methods:**

The secretome from human amniotic epithelial cells (AEC-SC) and human umbilical cord mesenchymal stem cells (UMSC-SC) was topically administrated on the back of imiquimod-induced psoriasis-like mice. Subsequently, we observed the skin lesions and skin inflammation of psoriasis-like mice. Next, we further analyzed the paracrine factors in AEC-SC and UMSC-SC by protein chips. Lastly, the effect of the crucial paracrine factor was investigated by imiquimod-induced psoriasis-like mice.

**Results:**

We found that AEC-SC had a better therapeutic effect on attenuating psoriasis-like skin lesions including skin scales, skin redness and skin thickness than UMSC-SC, and it had a better regulatory effect on keratinocyte hyperproliferation and altered differentiation. Thus, we focused on AEC-SC. Further study showed that AEC-SC reduced the infiltration of neutrophils and interleukin-17-producing T cells. Next, the analysis of AEC-SC with protein chip revealed that the levels of anti-inflammatory factor interleukin-1 receptor antagonist (IL-1ra) were much higher in AEC-SC compared to that in UMSC-SC. More importantly, the beneficial effect of AEC-SC on psoriasis-like skin lesions and skin inflammation of mice were significantly impaired when neutralizing with IL-1ra antibody, while the recombinant human IL-1ra showed a less protective effect than AEC-SC.

**Conclusions:**

The present study demonstrated that AEC-SC could efficiently ameliorate psoriasis-like skin lesions and skin inflammation and IL-1ra plays an essential role. Therefore, topical administration of AEC-SC may provide a novel strategy for treating psoriasis-like inflammatory skin diseases.

**Supplementary Information:**

The online version contains supplementary material available at 10.1186/s13287-022-03091-9.

## Background

Psoriasis is a common chronic inflammatory skin disease characterized by epidermal hyperplasia, increased angiogenesis, and prominent immune cell infiltration [1]. It affects 0.7% to 2.9% of the population in the world [2]. Psoriasis seriously affects patients' physical health, quality of life, and social interaction. Although the pathogenesis of psoriasis is not fully understood, compelling evidence suggests that the interplay among environmental factors, genetical susceptibility, skin barrier disruption and immune dysfunction play critical roles in the disease development [3]. Skin barrier is disrupted in psoriasis [4], which allows the entry of environmental substances into the dermis to induce immunological reactions and inflammation [5, 6]. Many inflammatory immune cells infiltrate the skin lesions of patients with psoriasis [7, 8]. These immune cells release inflammatory mediators and induce an immune cascade, which amplifies immune responses and further promote the disruption of the skin barrier and form a vicious circle between the skin barrier and immune cells [1, 9, 10]. Increasing evidence has illustrated the importance of the complex cross-talking between activated epidermal keratinocytes and infiltrating immune cells in the pathogenesis of psoriasis [7, 8]. Among inflammatory cytokines, interleukin-17A (IL-17A) plays a pivotal role in the pathogenesis of psoriasis [11, 12]. IL-17A induces excessive proliferation and abnormal differentiation of keratinocytes [12, 13]. And the activation and upregulation of IL17A in psoriatic skin produces a “feed forward” effect further contributing to sustain a vicious cycle of inflammation in psoriasis [10, 14]. It has been reported that targeting IL-23/IL-17 axis is a good target for psoriasis treatment [15].

Currently, there is no cure for psoriasis. From mild to modest psoriasis, topical steroids, phototherapy, conventional systemic agents such as ciclosporin, methotrexate, acitretin and other small molecules remain the mainstay of psoriasis therapy. However, long-term administration of these agents can cause some adverse effects, such as skin irritation, drug resistance and intolerance [1]. Therefore, it is necessary to investigate safe and effective treatments. Of note, mesenchymal stem cells (MSCs) have been reported to exhibit therapeutic effects on many immune-mediated diseases and inflammatory diseases in preclinical and clinical trials [16–19]. Meanwhile, epithelial-shaped human amniotic epithelial cells (hAECs) display profound immunomodulatory functions and inflammatory suppressive potential in experimental animal models, such as autoimmune uveitis [20], systemic lupus erythematosus [21], and experimental autoimmune encephalomyelitis [19]. But hAECs is rare reported in the treatment of inflammatory skin diseases. As for psoriasis, previous studies showed that administration of MSCs by subcutaneous or intravenous injection can ameliorate skin inflammation and skin lesion on psoriasis-like animal models by suppressing IL-17 producing γδ T cells or reducing Type I Interferon production by plasmacytoid dendritic cells [22–26]. However, in a clinical setting these administration routes are not easily acceptable for patients with psoriasis; therefore, the development of a clinically feasible stem cell-based administration method for the treatment of psoriasis is desirable.

Accumulated data indicate that paracrine factors secreted by stem cells facilitate cellular survival and regeneration [27, 28]. Moreover, paracrine factors can mediate the anti-inflammation and immunomodulatory function [29–32]. In some preclinical studies, Oksana et al. found that the secretome derived from MSCs reduces disease severity in inflammatory arthritis mice, enhances Treg function, and restores the ratio of Treg cells and Th17 cells [33]. Our recent study has demonstrated that the conditioned medium or the secretome derived from human amniotic epithelial cells (AEC-SC) attenuates experimental allergic conjunctivitis in mice [34]. However, the protective effect of secretome or paracrine factor derived from stem cells on psoriasis has not yet been reported. On the other hand, among different tissue-derived MSCs, human umbilical cord-derived MSCs (hUMSCs) are easily attained and have been widely used in psoriasis-like animal models [23, 25, 26]. Therefore, in the present study we compared the therapeutic effects of topical administration of AEC-SC and the secretome derived from human umbilical cord-derived MSCs (UMSC-SC) on imiquimod (IMQ)-induced psoriasis-like skin lesions in mice to find an efficient secretome. Furthermore, we also explored the underlying molecular mechanism and vital paracrine factor in the effective secretome.

## Materials and methods

### Isolation and culture of stem cells and collection of AEC-SC and UMSC-SC

hUMSCs and hAECs were isolated from human umbilical cord and human amnion membrane of term placenta from healthy women undergoing caesarean, respectively, according to previously described protocols [35, 36], and cultured in the α-MEM medium and DMEM/F12 medium, respectively, with 10% FBS and 1% Penicillin/Streptomycin (P/S) (all from Life Technology). The collection and subsequent use of adult tissues were approved by the Human and Animal Research Ethics Committee of Renji Hospital, School of Medicine, Shanghai Jiaotong University (license number KY2021-001). The people gave informed consent for sample collection.

For collection the UMSC-SC and AEC-SC, the cells were cultured in the α-MEM medium and DMEM/F12 medium, respectively, with 10% FBS at passage 3 at 90% confluency and washed with PBS for 3 times, then changed to basic medium DMEM/F12 and cultured for another 24 h, subsequently harvested the secretome and centrifuged the secretome at 2000 rpm/10 min at 4 °C to remove cell debris. We next used a BCA protein assay kit (Thermo Fisher) to measure the total protein concentration of UMSC-SC and AEC-SC and then normalized to the same concentration according to BCA levels of cell lysates for the following experiments. Lastly, the normalized UMSC-SC and AEC-SC were aliquoted and stored at − 80 °C for use.

### Flow cytometry

The culture cells were dissociated with trypsin and washed with cold PBS, then stained with IgG or monoclonal antibodies. The following is the information of antibodies: CD29-FITC, CD49f-FITC, CD73-FITC, CD105-APC, CD90-FITC, CD34-PerCP, CD31-FITC, CD45-FITC, and HLA-DR-FITC (all from eBioscience). Upon being washed with PBS, the cells were resuspended and at least 10^5^ events were acquired by using a BD Accuri™ C6 flow cytometer (BD bioscience).

As for immune cells derived from skin tissues of mice, skin tissues were cut into small pieces and digested with mixed enzymes containing 1 mg/ml collagenase (Sigma), 1 mg/ml hyaluronidase (Sigma) and 0.1 mg/ml DNase I (Roche) in a water bath shaker at 37 °C for 60 min. Then, the cell pellets were filtered and centrifuged, subsequently washed with PBS and resuspended in 1640 medium with 10% FBS, lastly stimulated with phorbol myristate acetate (PMA,100 ng/ml), Ionomycin (1 ug/ml) and Brefeldin A (10 µg/ml) at 37 °C incubator for 5 h. After stimulation, the cells were stained with CD3-FITC (eBioscience) and γδ TCR-PE-Cy7 (Biolegend), and fixed and permeabilized with the Cytofix/Cytoperm™ Kit (BD Biosciences). After that, the cells were stained with IL-17A-PE (BD bioscience). The labeled cells were resuspended and at least 10^5^ events were acquired by using BD Fortessa.

### Topical administration of the UMSC-SC or AEC-SC on the back skin of IMQ-induced psoriasis-like mice

8-week-old BALB/C female mice were purchased from Shanghai SLAC Laboratory Animal Co., Ltd (Shanghai, China). The mice were randomly divided into five groups with 6 mice for each group, and each mouse was kept in single cage under specific pathogen-free conditions. The groups were as follows: CON (blank control without adminstration of IMQ, DMEM/F12 basic medium pretreatment). IMQ (IMQ, DMEM/F12 basic medium), AEC-SC (IMQ, AEC-SC) and UMSCM (IMQ, UMSC-SC). The psoriasis-like skin inflammation model was induced by IMQ as previously described protocols with minor modification [12]. In brief, 62.5 mg Aldara IMQ cream (5%, 3.125 mg active compound, 3 M Pharmaceuticals) was smeared on the shaved back of BALB/c mice daily for consecutive 6 days. 0.5 ml AEC-SC or UMSC-SC or DMEM/F12 basic medium was topically applied by wet compress on the shaved back of mice twice daily from day 1 to day 6 before administration with IMQ. The mice were photographed on day 7. All mice were euthanized on day 7 and the back skin samples of mice were collected for further analysis.

### Histopathology and immunofluorescence analysis

As for histopathological analysis, the skin tissues of mice were fixed with 4% paraformaldehyde (PFA) and embedded in paraffin, then cut into sections with a thickness of 5 μm. The skin sections were stained with hematoxylin and eosin (H&E) and visualized with an inverted microscope.

As for immunofluorescence analysis, the skin tissues of mice were fixed with 4% PFA and embedded in OCT, then cut into sections with a thickness of 10 μm. The skin sections were incubated with the following primary antibodies: anti-Ki67 and anti-Gr-1 (Abcam, Cambridge). After washing with PBS, the skin sections were incubated with corresponding conjugated secondary antibodies. The slides were then visualized using an inverted fluorescence microscope. For qualification analysis, at least six representative sections for each group were counted. At least three mice were used in each group. Image J was used for image analysis.

### Analysis of soluble factors in the AEC-SC and UMSC-SC

To measure the soluble factors in the AEC-SC, a protein antibody array was performed with a Raybiotech L-series human Antibody Array 507 (Raybiotech). The expression levels of 507 human target proteins, including cytokines, chemokines, growth factors, angiogenic factors, soluble receptors, soluble adhesion molecules, and other proteins in the AEC-SC and UMSC-SC were simultaneously detected. The secretome from adult foreskin fibroblast (HEF-SC) was used as a control. The procedure was performed according to the manufacturer’s instructions. Lastly, the fluorescent signals on the glass slide were scanned with GenePix 4000B (Axon Instruments). For each array, the background was subtracted from the protein intensity values, and the values were scaled to the internal control and floored at 1 unit.

### ELISA analysis

The AEC-SC and UMSC-SC were collected and normalized to the same concentration according to BCA levels of cell lysates. The concentration of IL-1ra was measured using ELISA kit of IL-1ra (R&D) according to the manufacturer’s instruction. For ELISA kits, the assay range was 5–500 pg/ml.

### Culture and stimulation of HaCaT cells

HaCaT cells were cultured in DMEM medium supplemented with 10% FBS and 1% P/S and stimulated with 10 ng/ml TNFα for 15 min, then performed real-time PCR or western blot experiment. For AEC-SC pretreatment, HaCaT cells were incubated with AEC-SC for 4 h, then stimulated with 10 ng/ml TNFα for 15 min.

### Western blot analysis

The cells were lysed using RIPA Buffer (Beyotime). The protein concentration was determined by BCA kit. The PVDF membranes (Millipore) were blocked with 5% nonfat milk and incubated with primary antibodies (p-P65, p-P38, P65, P38, and b-Actin are from cell signaling technology) overnight at 4 °C. After washing with TBST, the PVDF membranes were incubated with corresponding HRP-conjugated secondary antibodies (Proteintech) for one hour at room temperature. Densitometric analysis of proteins was performed by Tanon 5200S (Tanon). b-Actin was used as internal control.

### Quantitative real-time PCR

The total RNA of cells was extracted using TRIzol reagent (Takara, Japan) and reverse transcribed into cDNA using the PrimeScript RT reagent kit (Takara). Real-time PCR (RT-PCR) was performed with SYBR Green PCR Master mix (Vazyme, China) and normalized by the expression of GAPDH. The relative amount of each gene was measured using the 2^−ΔΔCT^ method. All quantitative RT-PCR experiments were performed at least three independent experiments. For mice sample, the samples were harvested from at least three mice and the RT-PCR experiments were repeated for 3 times. The information of the primers was listed in Additional file 1: Table S1.

### Statistical analysis

The data are presented as mean ± SEM at least 3 independent experiments, and statistical analysis was assessed by SPSS software 22.0 and statistical significance were determined using Student’s *t*-test for comparison of three groups. For multiple comparisions, statistical significance was determined by one-way ANOVA with Tukey’s multiple comparisons test. *P* value less than 0.05 was considered significant.

## Results

### The therapeutic effect of AEC-SC on IMQ-induced psoriasis-like skin lesions in mice is better than UMSC-SC

hAECs and hUMSCs were isolated and cultured according to the protocol in the materials and methods section. The characteristics of hAECs were identified by flow cytometry. Additional file 2: Fig. S1 shows that hAECs expressed high levels of Epcam, CD49f, CD73 and CD29, low levels of CD31, CD34, CD45 and HLA-DR, while hUMSCs expressed high levels of CD105, CD90, CD73, CD44, CD29, low levels of CD31, CD34, CD45 and HLA-DR. The above results demonstrated that hAECs and hUMSCs had a high purity, indicating that the secretome from the hAECs and hUMSCs (AEC-SC and UMSC-SC) could be collected for the subsequent experiments. To harvest AEC-SC and UMSC-SC, the stem cells at passage 3 cultured in the correponding medium with 10% FBS at 90% confluency and washed with PBS, then changed to basic medium DMEM/F12 and cultured for 24 h, subsequently harvested the AEC-SC and UMSC-SC, respectively.

**Fig. 1:**
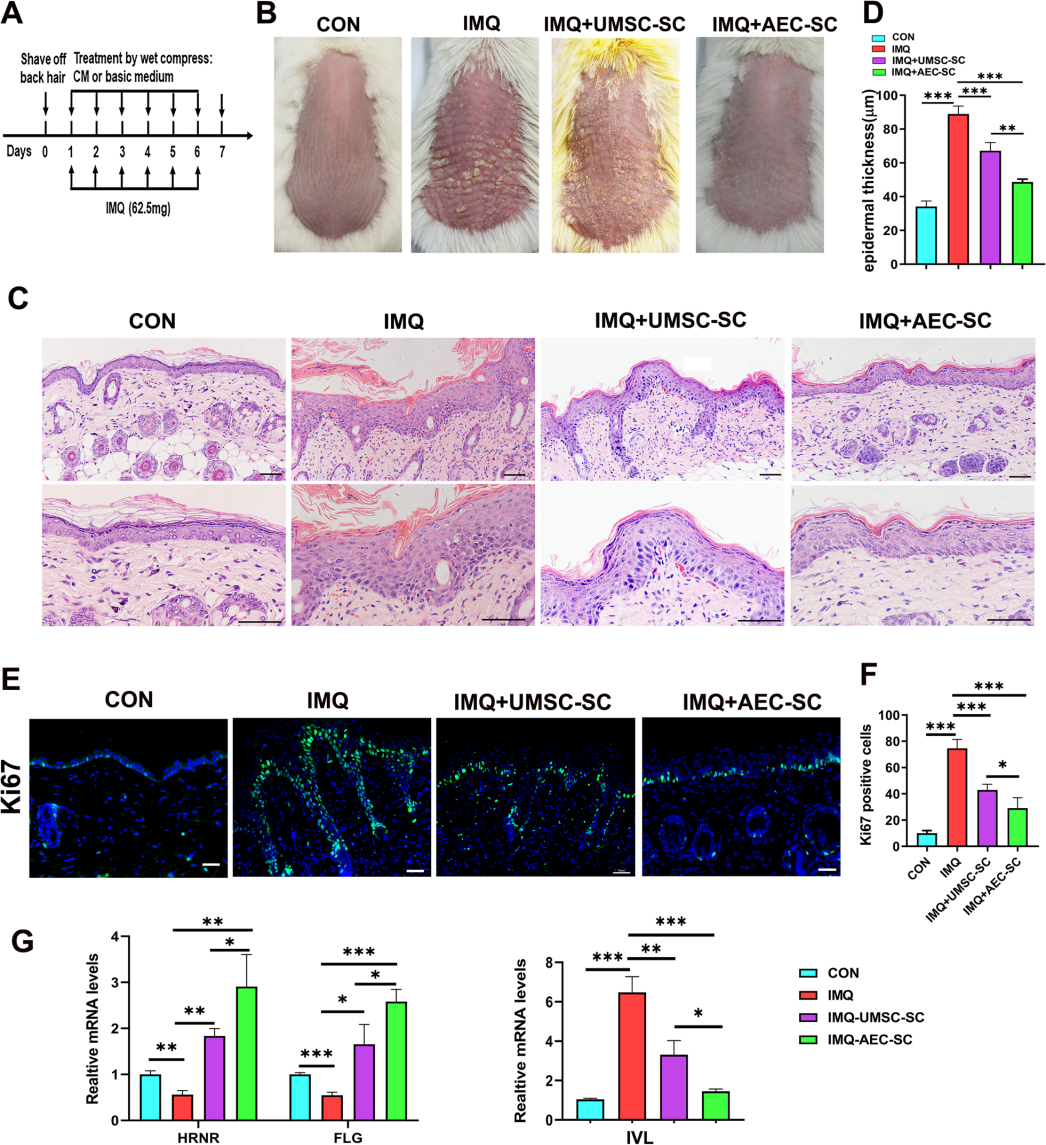
AEC-SC attenuated IMQ-induced psoriasis-like skin lesions. **a** A schematic illustration of animal experiment design. **b** Representative phenotypic image of mouse back skin after 6-day IMQ administration and different medium treatment. CON represents normal control mice. **c** Representative images of H&E staining of the back skin of mice in different groups. Scale bar: 50 μm. **d** Quantification of the epidermal thickness of mice in different groups. The data are expressed as mean ± SEM. At least six representative sections of the skin of mice were counted. At least three mice were used in each group. **P < 0.01, ***P < 0.001. **e** Immunostaining of back skin tissue of mice with Ki67 antibody and the representative images are shown. Scale bar: 50 μm. **f** Qualification of the number of Ki67 positive cells per field of the back skin of mice in different groups. **g** The relative mRNA levels of *FLG*, *HENR* and *IVL* in the back skin of mice in different groups. The mRNA levels of genes in CON group were set as 1. Data were collected from at least 3 mice and the data are expressed as the mean ± SEM, *P < 0.05, **P < 0.01, ***P < 0.001

To explore the therapeutic function of AEC-SC and UMSC-SC on psoriasis, we used an IMQ-induced psoriasis-like mouse model. In the secretome-treated groups, 0.5 ml AEC-SC or UMSC-SC was topically applied by wet compress on the shaved back of mice twice daily from day 1 to day 6 before IMQ treatment (Fig. 1a). The severity of skin lesions was assessed by skin scales, redness and thickness. Visible scales and redness aggravated in the IMQ group compared to the control group without IMQ treatment (Fig. 1b and Additional file 3: Fig. S2). However, AEC-SC treatment evidently reduced scales and redness (Fig. 1b and Additional file 3: Fig. S2**)**. H&E staining of the back skin of mice showed that epidermal thickness was increased in IMQ group but decreased in secretome-treated group, especially in AEC-SC (Fig. 1c, d and Additional file 4: Fig. S3). The above results indicated that AEC-SC attenuated psoriasis-like skin lesions.

Hyperproliferation with altered-differentiation of keratinocytes are the main characteristics of psoriasis [37]. The disrupted skin barrier promotes subsequent immune dysfunction, which contributes to the maintenance of inflammatory skin microenvironment of psoriasis [38]. To determine whether AEC-SC or UMSC-SC affect the integrity of skin barrier, we investigated the proliferation and differentiation of keratinocytes. Compared to the control group, IMQ  treatment increased Ki 67 expression in the epidermis of skin, however, AEC-SC reduced Ki-67 level, which is more than UMSC-SC (Fig. 1e and f). *Filaggrin (FLG), Hornerin (HRNR)* and *Involucrin (IVL)* are the late differentiation biomarkers of the epidermis [39, 40]. The expression of *FLG* and *HRNR* was decreased in the skin lesions of psoriatic patients [41, 42], while *IVL* expression was increased [43], which were also observed in the IMQ treated mice (Fig. 1g). However, AEC-SC treatment reversed their expression, which is better than UMSC-SC (Fig. 1g). Taken together, the above results indicated that AEC-SC can more efficiently inhibit the hyperproliferation of keratinocytes, regulate the altered differentiation of keratinocytes and restore skin barrier in IMQ-induced psoriasis-like mice than UMSC-SC. Therefore, we focused on AEC-SC in the following experiments.

### Topical administration of AEC-SC reduces the inflammation in IMQ-induced psoriasis-like mice

**Fig. 2:**
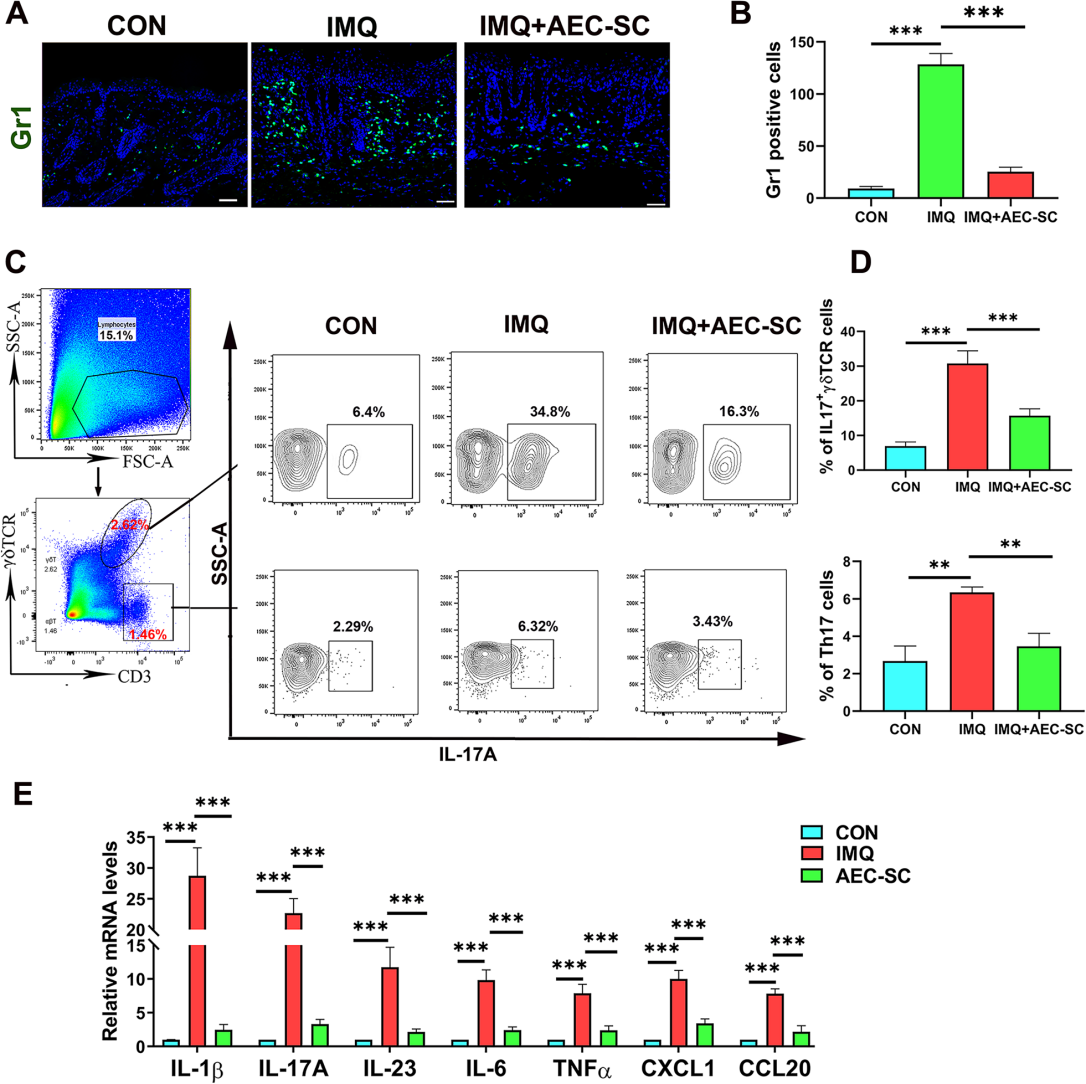
AEC-SC reduced the inflammation of back skin in IMQ-induced psoriasis-like mice. **a** Immunostaining of back skin tissue with a Gr-1 antibody and the representative images are shown. Scale bar: 50 μm. **b** Qualification of the number of Gr-1 positive cells of lesion skin tissue per field in different groups. At least 6 fields for each group were counted and at least three mice were used in each group. **P < 0.01, ***P < 0.001. **c** Flow cytometry analysis of IL7-producing δγ T cells (IL-17^+^ γδ T cells) and IL-17-producing γδ TCR negative cells (Th17 cells). **d** Quantification of IL-17^+^ δγ T cells and Th17 cells. **e** The relative mRNA levels of *IL-1β, IL-17A, IL-23*, *IL-6*, *TNFα, CXCL1*, and *CCL20* in the lesion skin of mice in each group. The mRNA levels of genes in CON group were set as 1 and the data are expressed as the mean ± SEM, *n* = 3. *P < 0.05, **P < 0.01, ***P < 0.001

To determine whether AEC-SC affects the local inflammatory environment in psoriasis-like dermatitis, we examined immune cell infiltration and the expression of cytokines and chemokines in skin lesion site of mice in various groups. Neutrophil infiltration in the skin lesions is a pathological feature of psoriasis [44]. Consistently, immunofluorescent staining of a Gr1 antibody showed a large number of neutrophils were in the dermis of skin lesion site of IMQ-treatment group, which was alleviated by AEC-SC treatment (Fig. 2a and b).

IL-17A has been demonstrated to play an essential role in the development and maintenance of psoriasis [10–13]. Th17 cells and dermal γδ T cells can produce IL-17A in psoriatic lesions [8, 45]. Previous studies reported that dermal γδ T cells are the main source of IL-17A in the skin lesions of IMQ-induced psoriasis-like mice [8, 12]. Consistent with these studies, our data shows that approximately 90% of IL-17A-producing cells in IMQ-induced mouse skin lesions came from γδ T cells (Additional file 5: Fig. S4). To determine if AEC-SC affects IL-17A producing, we analyzed IL17A-producing γδ T cells (CD3 + IL17A + γδ T cells) and Th17 cells (CD3 + IL17A + γδ TCR- cells) in the back skin of mice  by flow cytometry. Figure 2c and d shows that IL17 + δγ T cells and Th17 cells increased in IMQ group, decreased in AEC-SC group (Fig. 2c and d). In addition, AEC-SC inhibited the expression of proinflammatory cytokines, such as *IL1β*, *IL-17*A, *TNF-α*, *IL-23,* and *IL-6*, which are involved in psoriatic skin inflammation and all increased in the skin lesions of IMQ mice (Fig. 2e). The similar pattern was observed for chemokines CXCL1 and CCL20*,* which mediate neutrophil and IL-17-producing T cell recruitment, respectively (Fig. 2e). Taken together, the above results demonstrated that AEC-SC could reduce the infiltration of neutrophils and IL-17-producing T cells (Th17 and IL-17-producing γδ T cells), decrease the levels of inflammatory cytokines and chemokines, which lead to a reduction of skin inflammation in IMQ-treated mice.

### Anti-inflammatory factor IL-1ra is abundant in AEC-SC

**Fig. 3:**
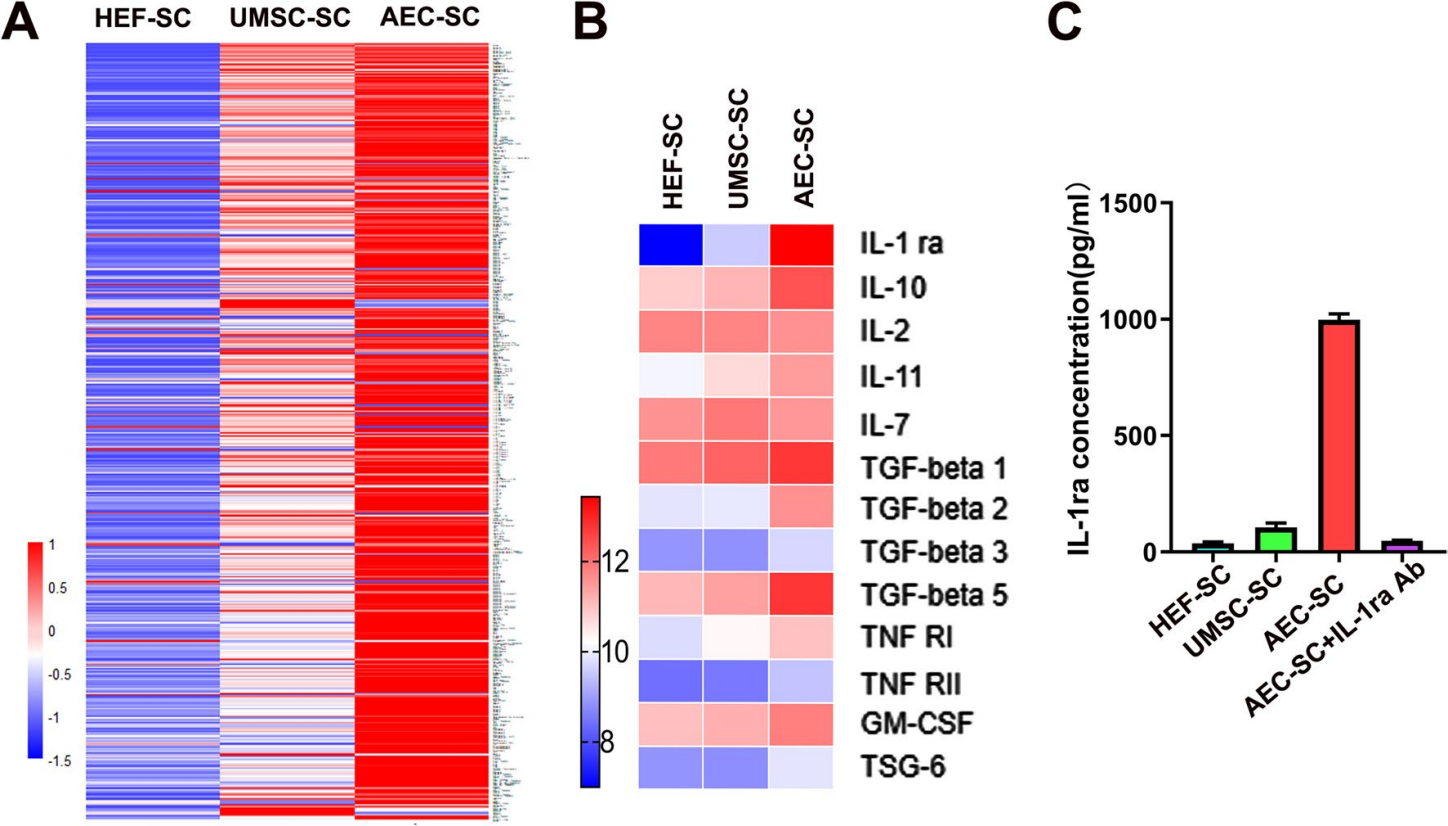
Analysis of paracrine factors in the AEC-SC and UMSC-SC. **a** Soluble factors in the AEC-SC and UMSC-SC were analyzed by a human antibody array 507. HEF-SC was used as a control. The normalized array data of 507 proteins in the secretome from different cells were analyzed by SAM, and the relative concentrations of these factors were shown as a “heat map”. **b** The relative concentrations of anti-inflammatory meditors were shown as a heat map. **c** ELISA analysis of IL-1ra concentration in the groups of HEF-SC, UMSC-SC, AEC-SC, and AEC-SC neutralized with IL-1ra antibody

The above results showed that the therapeutic effect of on IMQ-induced dermatitis in mice. To find the vital factor of AEC-SC in the process, we performed a protein chip assay of 507 protein factors including growth factors, cytokines, chemokines. The secretome derived from adult foreskin fibroblasts (HEF-SC) were used as a control. The relative expression levels of the soluble factors in the secretome are shown as a heat map in Fig. 3a. The paracrine factors in AEC-SC were generally higher than those in the UMSC-SC and HEF-SC (Fig. 3a, Additional file 6: Table S2). Because psoriasis is a chronic inflammatory disease with prominent immune cell infiltration and proinflammatory factors release in the skin of psoriasis [1, 9]. By focusing on the anti-inflammatory related factors, we found that the relative levels of IL-1 receptor antagonist (IL-1ra), IL-10 and TGF β, were higher in AEC-SC than those in UMSC-SC and HEF-SC (Fig. 3b, Additional file 7: Table S3). Among these anti-inflammatory factors, the level of IL-1ra is the highest (Fig. 3b, Additional file 7: Table S3). Of note, previous studies have demonstrated that IL1β is an essential cytokine for synergistically acting with IL-23 to stimulate IL-1R1^+^ γδ T cells to producing IL17 in skin inflammation [8, 46]. Hence, we inferred that IL-1ra, as a natural IL-1 receptor antagonist, which downregulate the activity of IL-1β, may be a crucial factor in mediating AEC-SC function on IMQ-induced skin inflammation. Next, we focused on IL-1ra in the subsequent experiments. ELISA assay showed that the concentration of IL-1ra in AEC-SC was up to 1000 pg/ml in AEC-SC, while that in UMSC-SC was only 105.6 ± 19.16 pg/ml. Thus, we applied exogenous human recombinant IL-1a with the concentration 1000 pg/ml in the following experiments. In addition, we verified that IL-1ra antibody (Ab) could neutralize IL-1ra in the AEC-SC (Fig. 3c) and IL-1ra Ab can be used in the following experiments. These data revealed that IL-1ra are abundant in AEC-SC, which may be involved in AEC-SC inhibitory effects on psoriasis-like mice.

### IL-1ra is necessary for AEC-SC to alleviate psoriasis-like dermatitis

**Fig. 4:**
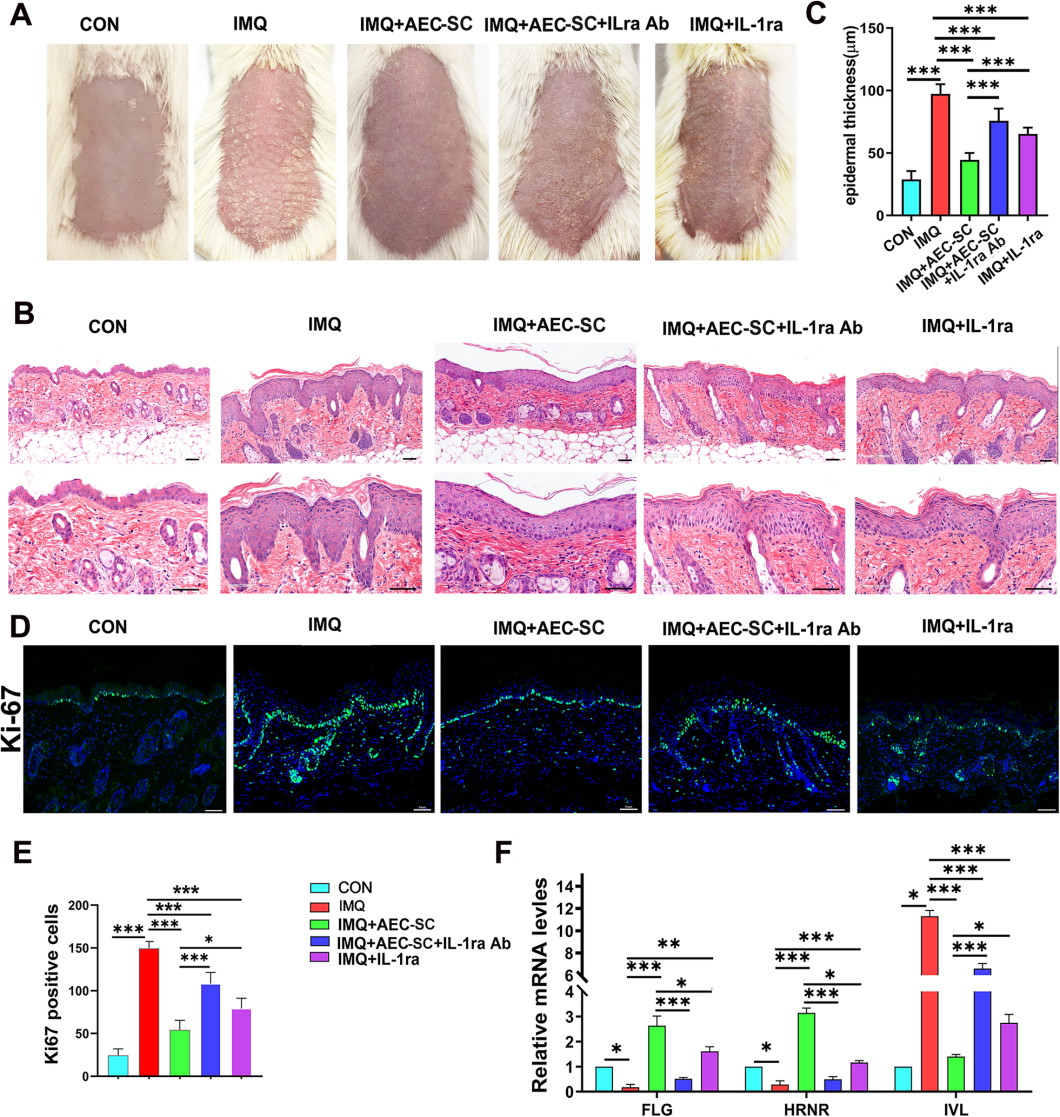
Anti-inflammatory factor IL-1ra is necessary for AEC-SC-mediated amelioration on skin lesions of IMQ-induced psoriasis-like mice. **a** Representative phenotypic image of mouse back skin  after 6-day IMQ administration and different medium treatment. **b** Representative image of H&E staining of the back skin of mice in different groups. Scale bar: 50 μm. **c** Quantification of epidermal thickness of mice in different groups. **d** Immunostaining of lesion skin tissue with Ki67 antibody and the representative images are shown. Scale bar: 50 mm. **e** Qualification of the number of Ki67 positive cells for per field of the lesion skin tissue in different groups. **f** Relative mRNA levels of *FLG*, *HENR* and *IVL* in the lesion skin tissue of mice in different groups. The mRNA levels of genes in CON group were set as 1 and the data are expressed as the mean ± SEM, *n* = 3. *P < 0.05, **P < 0.01, ***P < 0.001

Based on the above observations that the level of IL-1ra is high in AEC-SC. We proposed a hypothesis that IL-1ra is implicated in the inhibitory effects of AEC-SC-mediated psoriasis-like dermatitis attenuation. As shown in Fig. 4, the therapeutic effect of AEC-SC was impaired when adding IL-1ra neutralizing antibody, as evidenced by significantly increased scales and epidermis thickness (Fig. 4a–c). Application of human recombinant IL-1ra could partly mimic the effects of AEC-SC (Fig. 4a–c). Similar results were observed in the following analysis of the proliferation of keratinocytes (Fig. 4d and e). As for the differentiation of keratinocytes, AEC-SC countered IMQ-induced expression pattern of *FLG, HRNR and IVL* in the skin lesions (Fig. 4f). However, the effect of AEC-SC on regulating the altered differentiation of keratinocytes was significantly impaired after addition of IL-1ra neutralizing antibody in IMQ-treated mice (Fig. 4f). Of note, IL-1ra could partly mimic the effect of AEC-SC on the expression of *FLG*, *HRNR* and *IVL* (Fig. 4f).

**Fig. 5:**
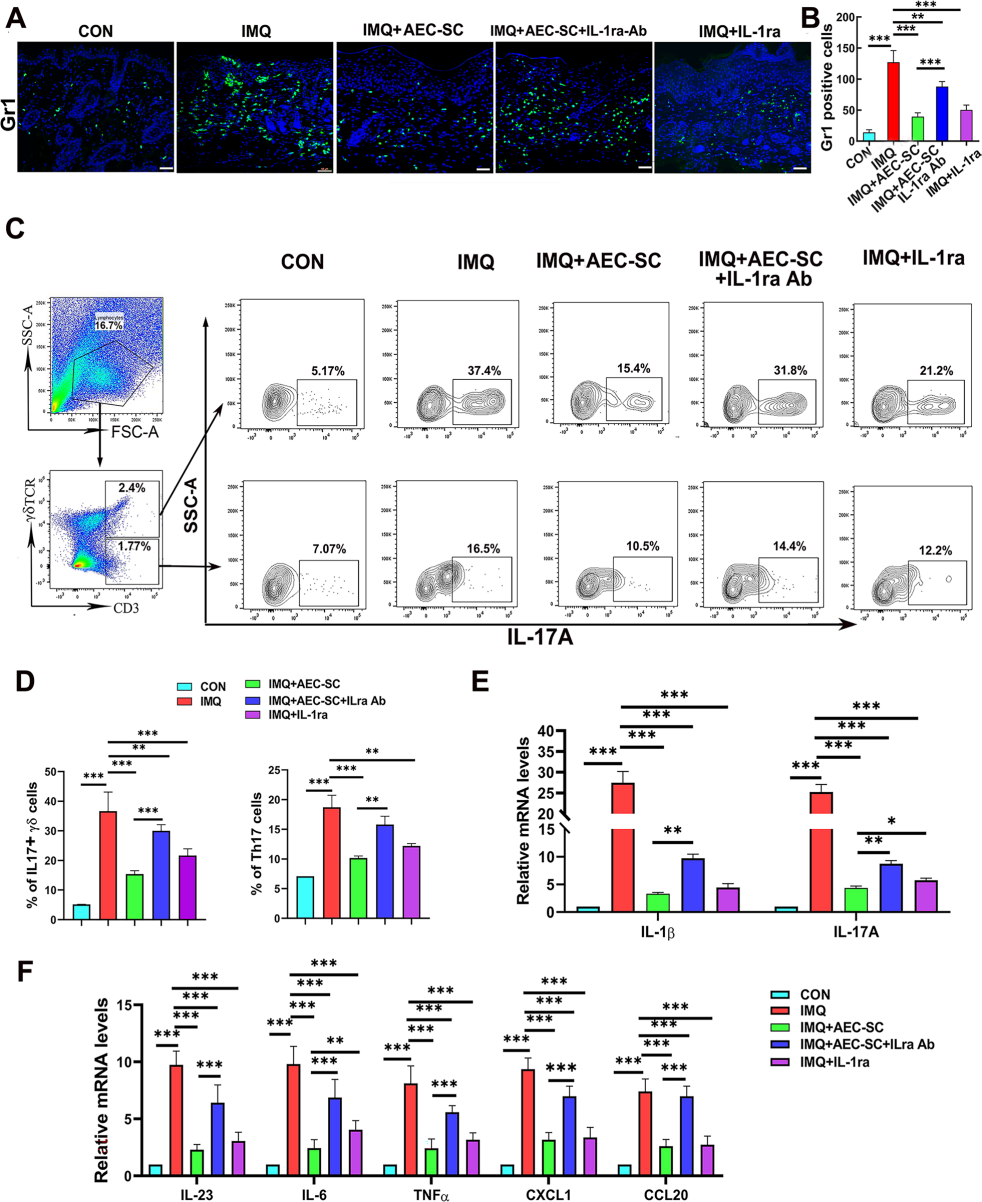
Anti-inflammatory factor IL-1ra is necessary for AEC-SC-mediated immunosuppression on skin inflammation of IMQ-induced psoriasis-like mice. **a** Immunostaining of back skin tissues with a Gr-1 antibody and the representative images are shown. Scale bar: 50 μm. **b** Qualification of the number of Gr-1 positive cells of back skin for per field in different groups. **c** Flow cytometry analysis of IL-17-producing γδ T cells and Th17 Cell. **d** Quantification of IL-17^+^ γδ T cells and Th17 cells. **e**, **f** The relative mRNA levels of *IL-1β, IL-17A, IL-23, TNF-a, IL-6, CXCL1* and *CCL20* in the lesion skin tissue of mice in each group by real-time PCR analysis. *P < 0.05, **P < 0.01 and ***P < 0.001

We next examined the inflammation reaction in the dermis of mice. As shown in Fig. 5, neutrophil, TH17 cells, and IL17-producing γδ T cells, as well as the expression of inflammatory mediators *IL-1β*, *IL-17A, IL-23*, *TNFα*, *IL-6*, *CXCL1* and *CCL20* in the back skin of IMQ-treated mice were increased. Such increases were all suppressed after AEC-SC treatment. Application of human recombinant IL-1ra alone yielded a less inhibitory effect than AEC-SC, and the effects of AEC-SC could be impaired by IL-1ra neutralizing antibody. Collectively, these data indicate that the beneficial effects of AEC-SC on psoriasis-like dermatitis may be mainly due to its higher levels of IL-1ra.

On the other hand, IL-1ra is abundant, thus we tested the infiltration and distribution of AEC-SC by immunofluorescence staining with human IL-1ra antibody, which has been demonstrated to be less cross-reactivity with mouse IL-1ra by manufacturer. Additional file 8: Fig. S5 shows that IL-1ra mainly remained in the dermal of AEC-SC group. In contrast, less signals were observed in the normal control group, IMQ group, and AEC-SC with human IL-1ra Ab group (Additional file 8: Fig. S5). The data indicate that AEC-SC could penetrate the skin barrier of patients with psoriasis to suppress the inflammation response in the skin microenvironment.

### AEC-SC reduces inflammatory response of HaCaT cells induced by TNFα

**Fig. 6:**
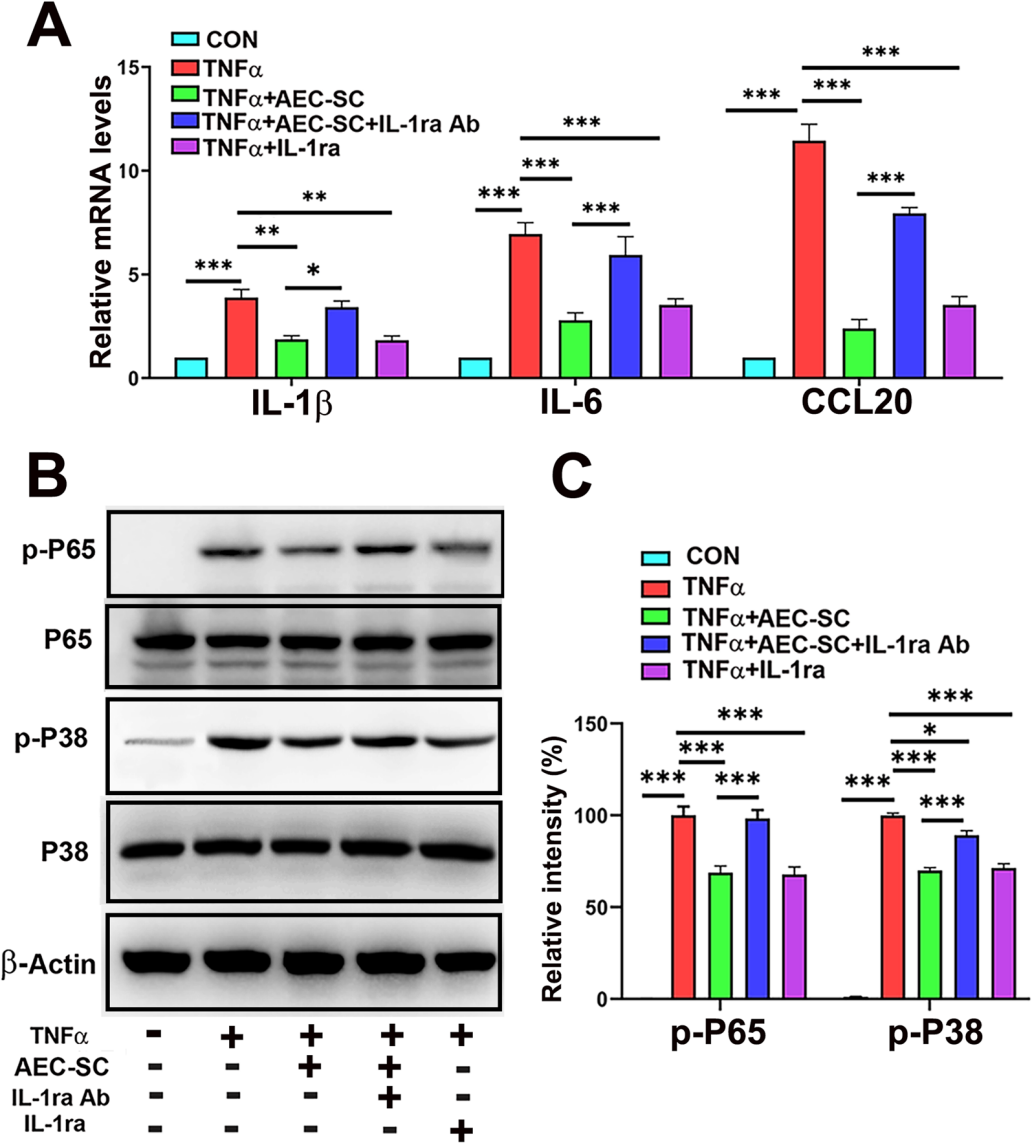
AEC-SC inhibited the inflammation stimulated by TNFα in HaCaT karatinocytes and IL-1ra mediated the function of AEC-SC. **a** The relative mRNA levels of *IL-1β, IL-6* and *CCL20* in HaCaT cells in each group as indicated. Data was collected from at least 3 separate experiments and the data are expressed as the mean ± SEM.*P < 0.05, **P < 0.01 and ***P < 0.001. **b** Western blot analysis of the protein levels of NF-κb p-P65 and MAPK p-P38 in the cell lysates of HaCaT cells. HaCaT cells were pretreated with AEC-SC or AEC-SC + IL-1ra Ab or IL-1ra for 4 h, then stimulated with TNFα for 15 min. **c** Qualification of relative protein levels of p-P65 and p-P38 in the cell lysates

Keratinocytes are the major components of the epidermis. Keratinocytes can produce many inflammatory mediators, which initiates aberrant immune responses. Because application with AEC-SC abolished the inflammation of IMQ-induced dermatitis and significantly decreased the expression of cytokines and chemokines, we hypothesized that AEC-SC might regulate these factors production in keratinocytes. We cultured HaCaT cells, a transformed human immortalized keratinocyte cell line [47], and stimulated with TNF-α. We found that AEC-SC could reduce the production of inflammatory mediators *TNFα*, *IL-1β* and *CCL20* of HaCaT cells stimulated by TNFα (Fig. 6a). However, IL-1ra neutralizing antibody abrogated the inhibitory effects of AEC-SC. Application of IL-1ra could reduce the expression of these inflammatory mediators. We next explored if AEC-SC affected the inflammatory pathways. As shown in Fig. 6b and c, NF-κb p65 and p38 MAPK were phosphorylated when HaCaT cells were stimulated with TNFα, AEC-SC downregulated the levels of p-P65 and p-P38, while IL-1ra neutralizing antibody eliminated the effects of AEC-SC. Human recombinant IL-1ra also inhibited the activation of NF-κb p65 and p38 MAPK pathway (Fig. 6b and c). These data suggested that AEC-SC could suppress the inflammatory response in HaCaT cells through inhibiting the activation of NF-κb and MAPK pathway and IL-1ra could mediate the process.

## Discussion

Increasing evidence shows that hAECs have immunomodulatory and anti-inflammatory effects in autoimmune and inflammatory diseases [20, 21, 28]. Paracrine function is believed to be an important therapeutic mechanism in mediating the function [29–32]. Psoriasis is a common inflammatory skin disease [1]. In the present study, we firstly demonstrated that topical administration of AEC-SC can efficiently alleviate IMQ-induced skin lesions (Fig. 1). AEC-SC inhibited the hyperproliferation and regulated the altered differentiation of keratinocytes, which benefited skin barrier restoration (Fig. 1d and e). AEC-SC attenuated skin inflammation through suppressing the infiltration of neutrophils and IL-17A-producing T cells and reducing the expression of inflammatory cytokines and chemokines. AEC-SC also decreased the production of IL-1β, IL-6, and CCL20 in the keratinocytes, and inhibited proinflammatory signaling pathways such as NF-κB and p38/MAPK pathways.

It has been proved that IL-23/IL17A axis plays a pivotal role in the pathogenesis of psoriasis. IL-17A in skin lesions mainly comes from dermal Th17 and IL-17-producing γδ T cells [8, 45]. Our study showed that AEC-SC reduced the infiltration of these IL-17-producing T cells in the skin. And more, AEC-SC inhibited CCL20 expression in the skin. CCL20 is the only chemokine in vivo to attract IL-17A-producing CCR6+ immune cells to the skin [10]. It may explain why AEC-SC alleviated the infiltration of Th17 and IL-17-producing γδ T cells. The similar pattern was seen on neutrophils accumulation and CXCL1 expression (Fig. 2). Meanwhile, AEC-SC also inhibited the expression of IL-1β and IL-23, which are essential for IL-17A production [8, 48]. Moreover, AEC-SC can also suppress the expression of other inflammatory factors IL-6 and TNFα (Fig. 2e), which are important for maintaining the inflammatory environment of psoriasis [49]. What’s more, these inflammatory cytokines induced over-proliferation and alter-differentiation of keratinocytes, contributing to the disruption of skin barrier [1, 9, 10]. Once the cytokines were eliminated, skin barrier would be restored, which have been demonstrated in our study.

As for the molecular mechanism of AEC-SC, we focused on IL-1ra, which was abundant in the AEC-SC. IL-1ra is a natural IL-1 receptor antagonist of IL-1α /IL-1β signals, which mediate a variety of inflammatory events. IL-1β is up-regulated in skin lesions of psoriatic patients [50]. IL-1β induces inflammation through activating neutrophils, helping inflammasome formation, and promoting IL-17A production. In addition, IL-1β is a relative “up-stream” cytokine. It promotes several proinflammatory cytokines and chemokines expression [51], contributing to the development of psoriasis [52]. IL-1ra is involved in suppressing the activation of M1 macrophages and dendritic cells, alleviating production of inflammatory cytokines TNF-α, IL-1β, IL-6, IL-12, and IL-23, and preventing the expansion of Th1 and Th17 cells in the injured skin. IL-1ra knock out BALB/C mice develop psoriasis-like cutaneous inflammation [53]. In our study, we also demonstrated that pretreatment of AEC-SC with specific neutralizing antibody for IL-1ra significantly abolished its therapeutic effects (Figs. 4, 5, 6). Interestingly, Anakinra, a recombinant human non-glycosylated homology of IL-1ra, has been approved effective in plaque psoriasis [54]. However, Anakinra should be injected subcutaneously, which is inconvenient for patients. A higher dose may cause allergic reaction or infection [55]. Therefore, AEC-SC, which can be topically applied, may be easily acceptant and causes less adverse reaction, and may be considered for the treatment of psoriasis-like diseases or other inflammatory skin diseases.

It is worthy to point out that the therapeutical effect of AEC-SC on psoriasis-like mice is better than that of IL-1a alone, which need further study. We think that other anti-inflammatory mediators, such as IL-10, TGF-β, could also contribute the effect. Therefore, AEC-SC may be superior to a single antibody or anti-inflammation drug.

There are many additional advantages to apply this hAECs for therapeutic purposes. First, hAECs are isolated from discarded term placenta [56]. Thus, they are easily available, do not need invasive procedures for harvesting and cause less ethical problem. In addition, hAECs possess good proliferation ability and activity [57]. It is possible to get a large number of hAECs for clinical administration. Secondly, preclinical trials and clinical trials have demonstrated that hAECs are safe, they do not cause allergic reaction, immune reaction, or tumor formation [58]. Third, AEC-SC is a non-living cells biological agent, which is easy to prepare, preserve, safe and easy to control its quality. Fourth, AEC-SC can penetrate skin barrier of the patients with psoriasis. Therefore, it could topically be administrated on patients with psoriasis.

## Conclusions

In the present study, for the first time, we demonstrated that AEC-SC inhibited the production of cytokines and chemokines, the infiltration of neutrophils and IL-17-producing T cells in the skin lesions, and the activation of inflammatory-related signaling pathway in the keratinocytes through IL-1ra-dependent mechanisms. These findings provide compelling evidence that AEC-SC restored the skin barrier and inhibited skin inflammation in the IMQ-induced psoriasis-like mice model. It supported that AEC-SC might be topically administrated to patients with psoriasis. Therefore, AEC-SC may provide a new clinical option to treat psoriasis and other inflammatory skin diseases.

## Supplementary Information

**Additional file 1. Table S1:** Primers used for real-time PCR.

**Additional file 2. Fig. S1:** Characterization of hAECs and hUMSCs by flow cytometry with antibody against Epcam, CD49f, CD90, CD105, CD73, CD29, CD44, CD31, CD45 and HLA-DR. **a** hAECs. **b** hUMSCs.

**Additional file 3. Fig. S2:** Phenotypic images of mouse back skin after 6-day IMQ administration and different medium treatment in various groups. CON represents normal control mice. At lease 4 mice were shown in each group.

**Additional file 4. Fig. S3:** Representative images of H&E staining of the back skin of mice in different groups. Scale bar: 50 μm. At least 4 mice were shown in each group.

**Additional file 5. Fig. S4:** Flow cytometry analysis of lesion skin tissues in IMQ-induced mice with mouse IL 17A and δγ TCR antibodies.

**Additional file 6. Table S2:** A table of normalized fluorescence signal intensity of various proteins in the HEF-SC, UMSC-SC, AEC-SC.

**Additional file 7. Table S3:** A table of normalized fluorescence signal intensity of anti-inflammatory related mediators in the HEF-SC, UMSC-SC, AEC-SC.

**Additional file 8. Fig. S5:** Immunostaining of lesion skin tissue of mice with human IL-1ra antibody in different groups and the representative images are shown. Scale bar: 50 μm. White arrows represent IL-1ra signals in the dermis in the skin.

## Data Availability

All related data and materials are available under request.

## Abbreviations

hAECs: Human amniotic epithelial cells
AEC-SC: The secretome from human amniotic epithelial cells
MSCs: Mesenchymal stem cells
hUMSCs: Human umbilical cord mesenchymal stem cells
UMSC-SC: The secretome from human umbilical cord mesenchymal stem cells
HEF-SC: The secretome from adult foreskin fibroblast
IL-1ra: Interleukin-1 receptor antagonist
IL-17A: Interleukin-17A
IL-12: Interleukin-12
IL-23: Interleukin-23
TNF: Tumor necrosis factor
IMQ: Imiquimod
H&E: Hematoxylin and eosin
FLG: Filaggrin
HRNR: Hornerin
IVL: Involucrin
