# Supplementary figures and images for: Topical administration of the secretome derived from human amniotic epithelial cells ameliorates psoriasis-like skin lesions in mice
Source: Stem Cell Res Ther. 2022 Aug 3;13:393. doi: 10.1186/s13287-022-03091-9 (PMC9351215; doi:10.1186/s13287-022-03091-9)

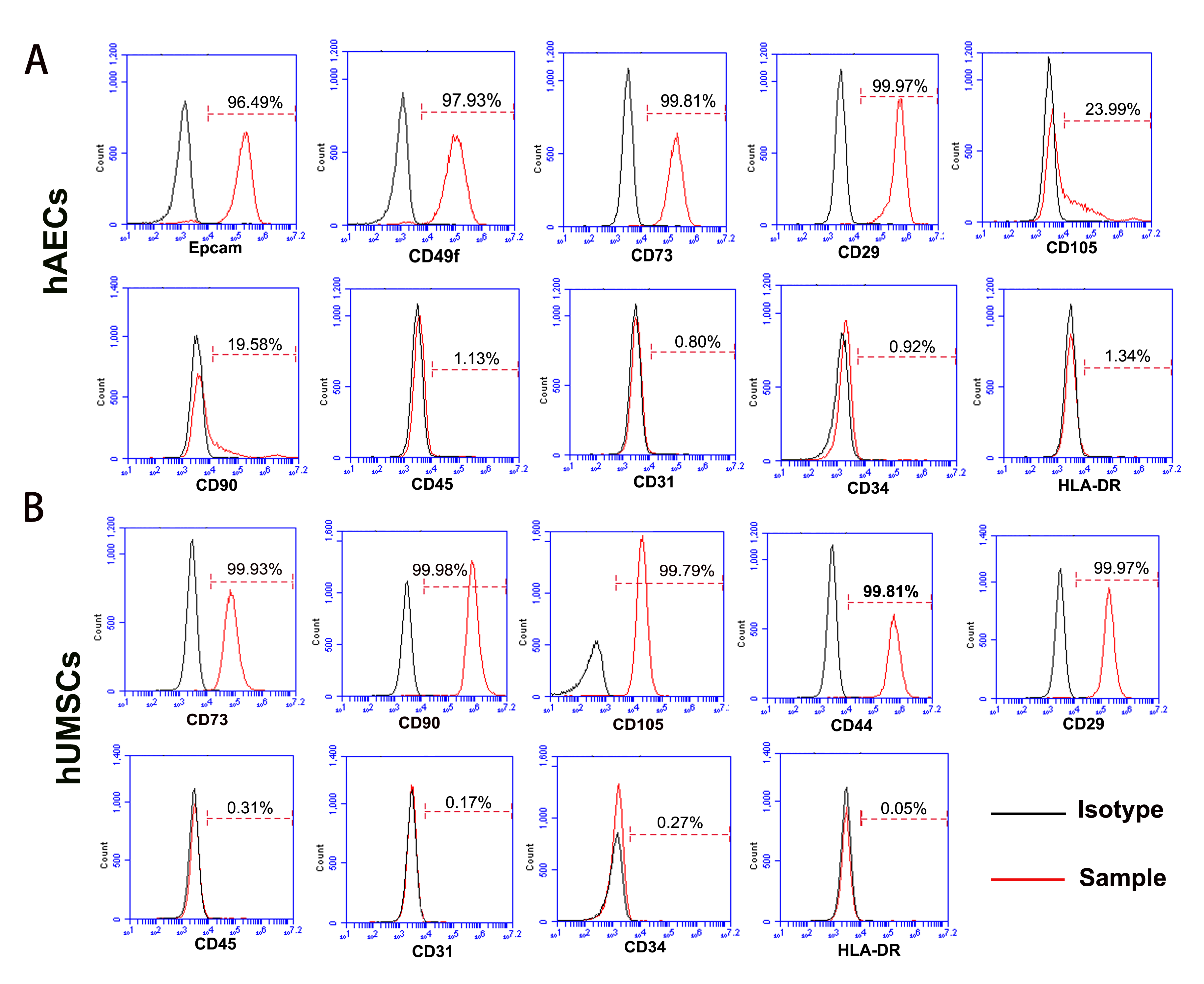

Supplement: Supplementary file 2 — Additional file 2. Fig. S1: Characterization of hAECs and hUMSCs by flow cytometry with antibody against Epcam, CD49f, CD90, CD105, CD73, CD29, CD44, CD31, CD45 and HLA-DR. a hAECs. b hUMSCs. [file 13287_2022_3091_MOESM2_ESM.tif]

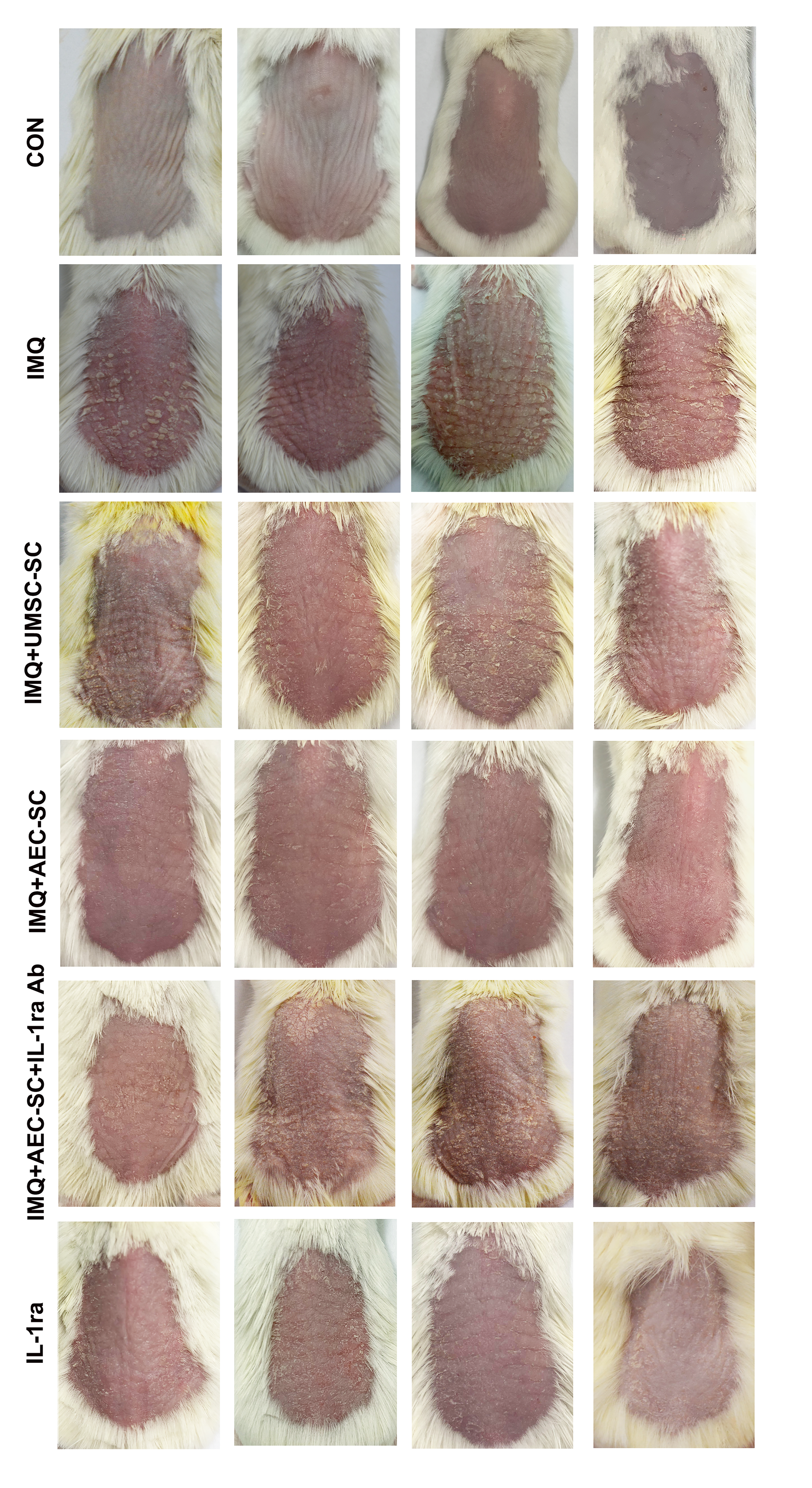

Supplement: Supplementary file 3 — Additional file 3. Fig. S2: Phenotypic images of mouse back skin after 6-day IMQ administration and different medium treatment in various groups. CON represents normal control mice. At lease 4 mice were shown in each group. [file 13287_2022_3091_MOESM3_ESM.tif]

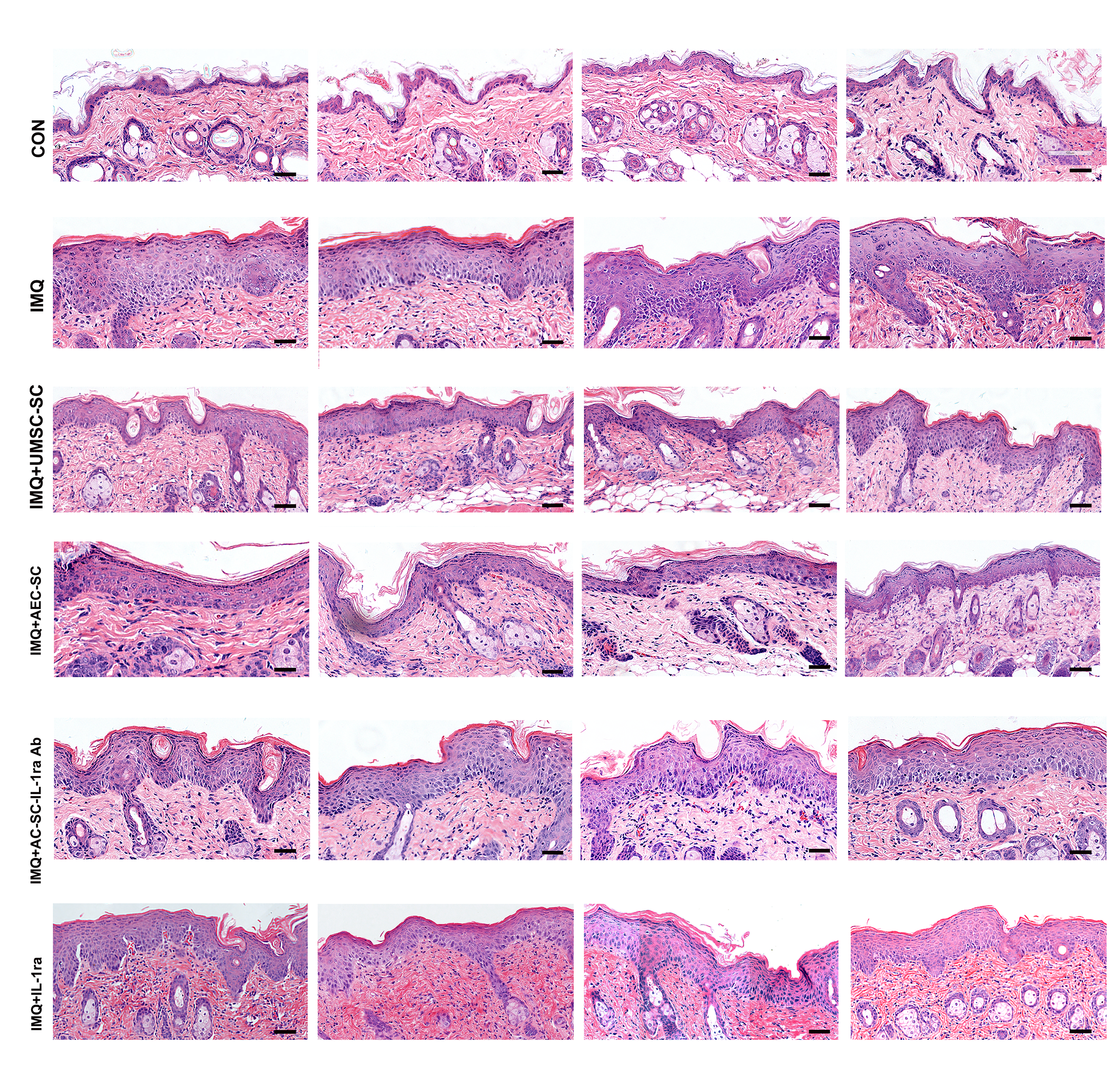

Supplement: Supplementary file 4 — Additional file 4. Fig. S3: Representative images of H&E staining of the back skin of mice in different groups. Scale bar: 50 μm. At least 4 mice were shown in each group. [file 13287_2022_3091_MOESM4_ESM.tif]

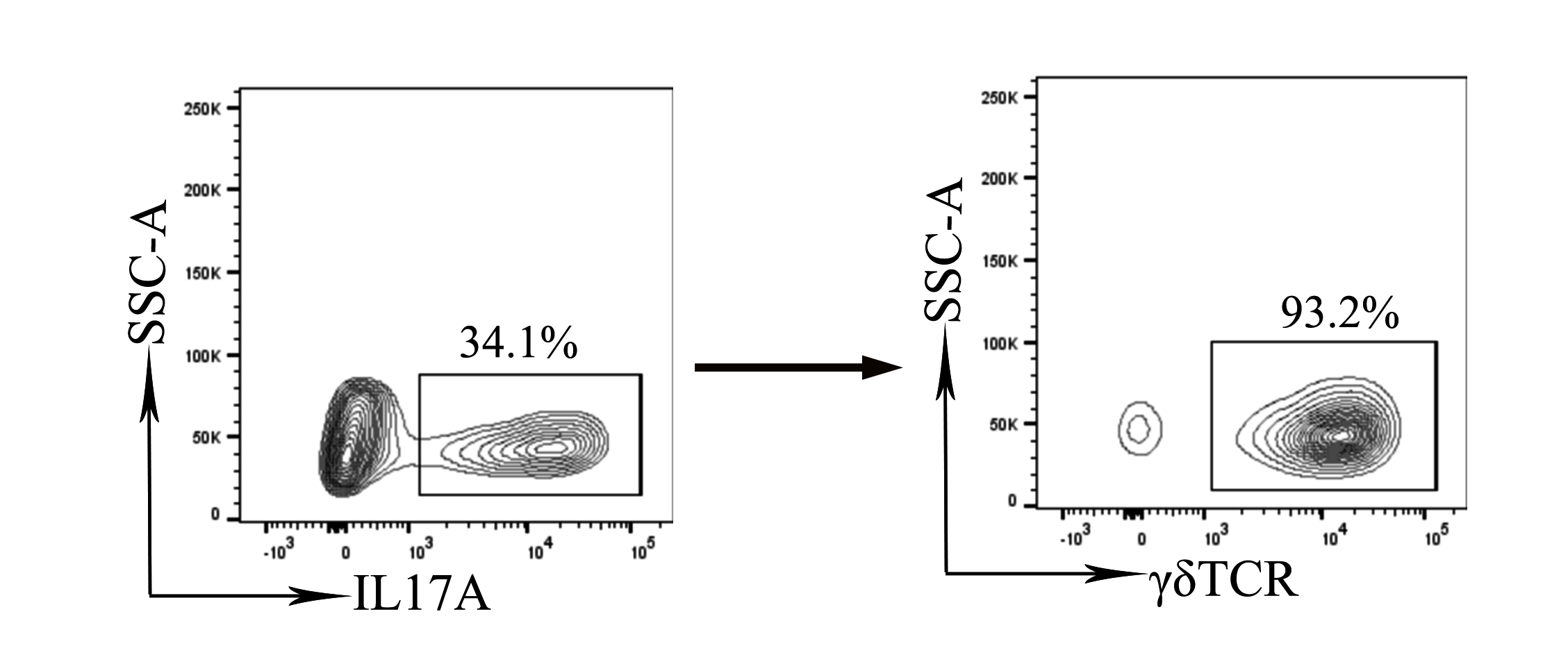

Supplement: Supplementary file 5 — Additional file 5. Fig. S4: Flow cytometry analysis of lesion skin tissues in IMQ-induced mice with mouse IL 17A and δγ TCR antibodies. [file 13287_2022_3091_MOESM5_ESM.tif]

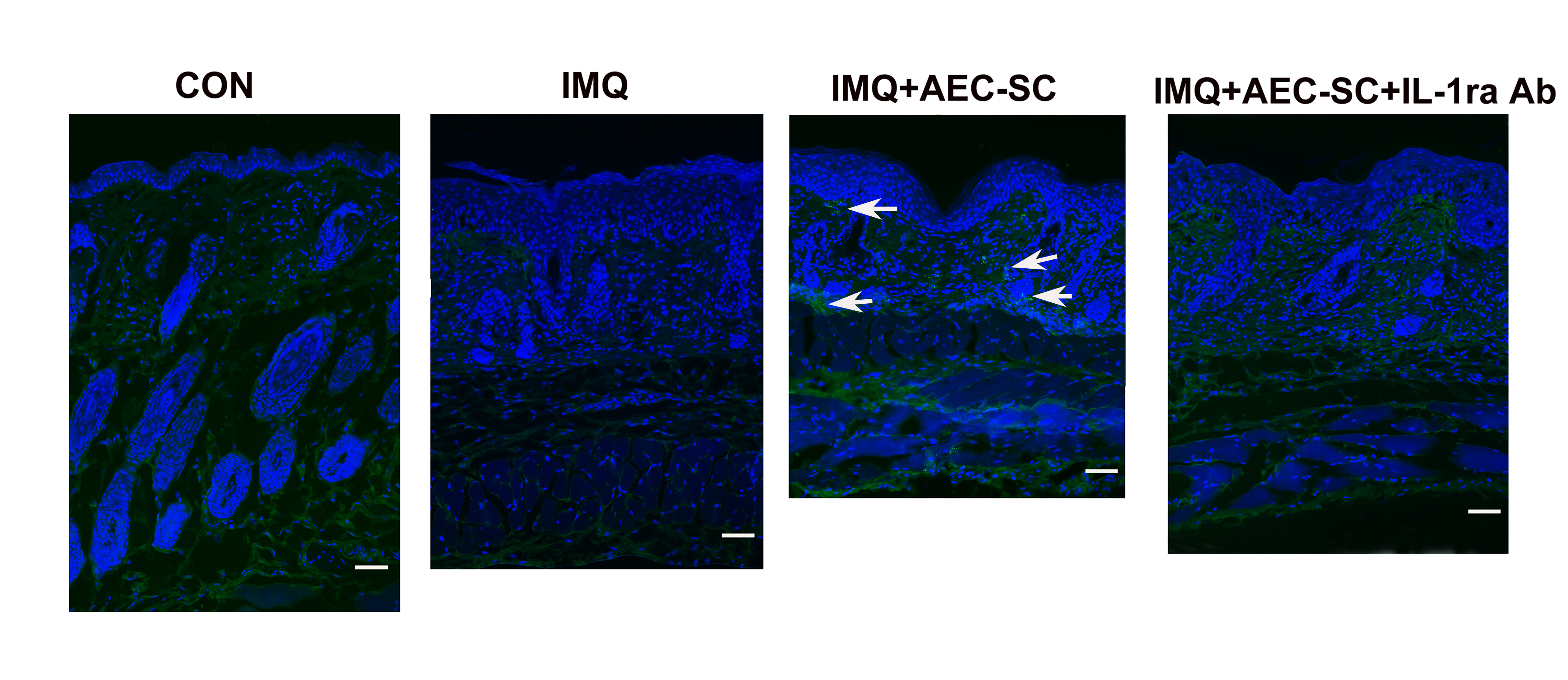

Supplement: Supplementary file 8 — Additional file 8. Fig. S5: Immunostaining of lesion skin tissue of mice with human IL-1ra antibody in different groups and the representative images are shown. Scale bar: 50 μm. White arrows represent IL-1ra signals in the dermis in the skin. [file 13287_2022_3091_MOESM8_ESM.tif]
